# Cerebrospinal fluid amyloid-β and cerebral microbleed are associated with distinct neuropsychiatric sub-syndromes in cognitively impaired patients

**DOI:** 10.1186/s13195-024-01434-7

**Published:** 2024-04-03

**Authors:** Qingze Zeng, Yanbo Wang, Shuyue Wang, Xiao Luo, Kaicheng Li, Xiaopei Xu, Xiaocao Liu, Luwei Hong, Jixuan Li, Zheyu Li, Xinyi Zhang, Siyan Zhong, Zhirong Liu, Peiyu Huang, Yanxing Chen, Minming Zhang, for behalf of Alzheimer’s Disease Neuroimaging Initiative

**Affiliations:** 1 Department of Radiology, Zhejiang University School of Medicine Second Affiliated Hospital, Shangcheng District, No.88 Jiefang Road, Hangzhou, 310009 China; 2 Department of Neurology, Zhejiang University School of Medicine Second Affiliated Hospital, Shangcheng District, No.88 Jiefang Road, Hangzhou, 310009 China; 3 Department of Neurology, Xinhua Hospital of Zhejiang Chinese Medical University, Hangzhou, China

**Keywords:** Alzheimer’s disease, Neuropsychiatry, Amyloid, Small vessel disease, Biomarkers

## Abstract

**Background:**

Neuropsychiatric symptoms (NPS) are prevalent in cognitively impaired individuals including Alzheimer’s disease (AD) dementia and mild cognitive impairment (MCI). Whereas several studies have reported the associations between NPS with AD pathologic biomarkers and cerebral small vessel disease (SVD), but it remains unknown whether AD pathology and SVD contribute to different sub-syndromes independently or aggravate same symptoms synergistically.

**Method:**

We included 445 cognitively impaired individuals (including 316 MCI and 129 AD) with neuropsychiatric, cerebrospinal fluid (CSF) biomarkers (Aβ42, p-tau, and t-tau) and multi-model MRI data. Psychiatric symptoms were accessed by using the Neuropsychiatric Inventory (NPI). Visual assessment of SVD (white matter hyperintensity, microbleed, perivascular space, lacune) on MRI images was performed by experienced radiologist. Linear regression analyses were conducted to test the association between neuropsychiatric symptoms with AD pathology and CSVD burden after adjustment for age, sex, education, apolipoprotein E (APOE) ε4 carrier status, and clinical diagnosis.

**Results:**

The NPI total scores were related to microbleed (estimate 2.424; 95% CI [0.749, 4.099]; *P* =0.005). Considering the sub-syndromes, the hyperactivity was associated with microbleed (estimate 0.925; 95% CI [0.115, 1.735]; *P* =0.025), whereas the affective symptoms were correlated to CSF level of Aβ_42_ (estimate -0.006; 95% CI [-0.011, -0.002]; *P* =0.005). Furthermore, we found the apathy sub-syndrome was associated with CSF t-tau/Aβ_42_ (estimate 0.636; 95% CI [0.078, 1.194]; *P* =0.041) and microbleed (estimate 0.693; 95% CI [0.046, 1.340]; *P* =0.036). In addition, we found a significant interactive effect between CSF t-tau/Aβ_42_ and microbleed (estimate 0.993; 95% CI [0.360, 1.626]; *P* =0.019) on severity of apathy sub-syndrome.

**Conclusion:**

Our study showed that CSF Aβ_42_ was associated with affective symptoms, but microbleed was correlated with hyperactivity and apathy, suggesting the effect of AD pathology and SVD on different neuropsychiatric sub-syndromes.

**Supplementary Information:**

The online version contains supplementary material available at 10.1186/s13195-024-01434-7.

## Introduction

Neuropsychiatric symptoms (NPS) are prevalent in cognitively impaired individuals including Alzheimer’s disease (AD) dementia and mild cognitive impairment (MCI). It has been demonstrated that the presence of NPS is associated with faster cognitive decline [1], worse prognosis [2], and lower quality of life [3]. According to the consistent associations of specific symptoms, some NPS could be consolidated into a kind of potential sub-syndromes [4, 5]. Based on a large sample of AD patients, the European Alzheimer’s Disease Consortium proposed four kinds of neuropsychiatric sub-syndromes: hyperactivity, psychosis, affective symptoms, and apathy [6]. However, the neurobiological basis of NPS is still unclear.

A growing body of research examined the associations between NPS and AD-related biomarkers [7–10]. Specifically, several studies have found that amyloid deposition was associated with higher depressive symptoms [10–12]. In addition, investigators using longitudinal cohort have observed that Aβ pathology may drive the development of apathy and anxiety [9, 11, 13]. Also, a recent review has concluded that NPS are associated with SVD [14]. For example, the presence of multiple microbleed or the severity of white matter hyperintensities (WMH) are related to high NPS burden [15–17]. Thus, a potential mechanism is that the NPS could be the consequence of AD pathology or vascular dysfunction. But it remains unknown about specific contribution of AD pathology and SVD on NPS in cognitively impaired individuals respectively.

In this study, we aimed to explore the effect of AD pathology and SVD on psychiatric symptoms in cognitively impaired individuals. Based on previous findings, we hypothesized that AD pathology and SVD could contribute to different sub-syndromes independently and aggravate same symptoms synergistically.

## Methods and materials

### Study population

All data used in the current study were from the ADNI database (http://adni.loni.usc.edu/). This ongoing project was launched in 2003 to develop clinical, neuropsychological, and neuroimaging biomarkers for early disease detection and progression monitoring of AD.

The inclusion criteria of participants as follows: 1) subjects diagnosed with MCI or AD; 2) each subject had undergone neuropsychological and neuropsychiatric assessment as well as cerebrospinal fluid (CSF) biomarkers examination and multi-model MRI scan. According to the inclusion criteria, we included totally 445 participants (including 316 MCI and 129 AD) from ADNIGO/2 dataset.

According to the ADNI Procedures Manual (https://adni.loni.usc.edu/wp-content/uploads/2008/07/adni2-procedures-manual.pdf), the ADNI criteria for MCI were: 1) subjective memory complaints; 2) objective memory loss defined as scoring below an education-adjusted cut-off score on delayed recall of the Wechsler Memory Scale-Revised (WMS-R) logical memory test II subscale: ≤ 11 for 16 or more years of education, ≤ 9 for 8-15 years of education c. ≤ 6 for 0-7 years of education (the maximum score is 25); 3) a global Clinical Dementia Rating (CDR) score of 0.5, memory box score must be at least 0.5; 4) a Mini-Mental State Examination (MMSE) score of equal to, or higher than 24 (the maximum score is 30); and 5) general cognitive and functional performance sufficiently preserved such that a diagnosis of dementia could not be made by the site physician at the time of screening [18].

The ADNI criteria for AD were: 1) subjective memory concern; 2) abnormal memory function documented by scoring within the education-adjusted ranges on the WMS-R logical memory II: ≤ 8 for 16 or more years of education, ≤ 4 for 8-15 years of education c. ≤ 2 for 0-7 years of education (the maximum score is 25); 3) MMSE score between 20 and 26 (inclusive); 4) CDR ≥ 0.5; 5) met the NINCDS/ADRDA criteria for probable AD [18].

### Neuropsychological assessment

Each participant has undergone neuropsychological examination including the MMSE, Montreal Cognitive Assessment (MoCA), Auditory Verbal Learning Test (AVLT), Logical Memory Test, Clock Drawing Test (CDT), Trial-Making Test-A (TMT-A), Trial-Making Test-B (TMT-B), Category Fluency Test, and Boston Naming Test (BNT) [18]. More details of the ADNI procedures of the cognitive assessment are publicly available on the website (www.loni.ucla.edu/ADNI).

### Neuropsychiatric assessment

In this study, psychiatric symptoms were accessed by using the Neuropsychiatric Inventory (NPI), which is a structural interview for estimating behavioral and psychological symptoms including delusion, hallucinations, agitation, depression, anxiety, elation, apathy, disinhibition, irritability, aberrant motor behavior, night-time behavior, appetite [19–21]. Each domain contains four items: presence, severity, frequency, and care-giver distress. The score of each domain (0-12 points) was calculated by symptom frequency (0-4 points) multiplied by severity (0-3 points). Total scores range from 0 to 144 [19].

According to suggestions of the European Alzheimer’s Disease Consortium, we divided all psychiatric symptoms into four subsyndromes: (1) hyperactivity (agitation, elation, disinhibition, irritability, and aberrant motor behavior); (2) psychosis (delusion, hallucinations, and night-time behavior); (3) affective symptoms (anxiety and depression); (4) apathy (apathy and appetite) [6, 22].

### Cerebrospinal fluid samples and quantification

CSF data were obtained by file “UPENNBIOMK_MASTER.csv” downloaded from the ADNI database. Levels of Aβ_42_, total tau (t-tau) and tau phosphorylated at threonine 181 (p-tau_181_) were measured using the multiplex 8 Luminex platform as previously described [23].

### Imaging acquisition

MRI data were acquired with the uniform ADNIGO/2 scan acquisition protocol as described by Jack et al [24], including 3D-T1, axial T2-FLAIR, and axial T2* sequence. For the detailed parameters of MRI acquisition please see https://adni.loni.usc.edu/methods/documents/mri-protocols/.

### Assessment of small vessel disease

SVD imaging markers included WMH, enlarged perivascular spaces (EPVS), microbleed, and lacunes. Visual assessment of SVD was performed by an experienced radiologist from our study team (Xiao Luo), blinding to the individual clinical diagnosis and neuropsychological data. WMH in periventricular regions and deep white matter were visual rated on T2-FLAIR images according to the 4-point Fazekas standard [25]. Severity of EPVS in basal ganglia (BG) and centrum semiovale (CSO) were assessed on T1-weighted images and rated as follow: 0 = none, 1 = 1-10, 2 = 11-20, 3 = 21-40 and 4 ≥ 40 EPVS 0 in any single slice, according to the previously proposed rating scale (https://www.ed.ac.uk/files/imports/fileManager/epvs-rating-scale-user-guide.pdf). Cerebral microbleeds were manifested as small, homogeneous round foci of hypointense on T2* images. Lacunes were referred to rounded or oval lesions, in the centrum semiovale, basal ganglia, internal capsule, or brainstem, with diameters between 3 mm and 20 mm, of CSF signal intensity on FLAIR images and high signal intensity on the rim. The microbleed and lacunes were defined as present (≥1 microbleeds or lacunes) or absent (no microbleeds or lacunes).

In order to further explore the potential effect of WMH volume, we downloaded the data of WMH volume segmented by Bayesian approach from ADNI dataset (ADNI_UCD_WMH_05_02_22_16Jan2024.csv) [26, 27].

### Statistical analysis

Statistical analyses were performed using IBM SPSS Statistics Version 24 (IBM SPSS Statistics for Windows). Results with *p* <0.05 were considered statistically significant.

Regarding demographic and clinical data, between-group differences were estimated by using Mann-Whitney U-test and Chi-square test. Continuous variables were expressed as mean ± standard deviation, and categorical variables were expressed as median (Q1, Q3) or percentage respectively

Linear regression analyses were conducted to test the association between neuropsychiatric symptoms with AD pathology and CSVD burden. We assigned the CSF-derived biomarkers (Aβ_42_, p-tau_181_, t-tau, p-tau_181_/Aβ_42_, t-tau/Aβ_42_) and CSVD visual rating scores (PV-WMH, DM-WMH, BG-PVS, CSO-PVS, microbleed, and lacunes) as independent variables, and considered NPI total score as dependent variable. In order to explore effect on specific subsyndromes, we further repeated the linear regression analyses with NPI score of four subsyndromes as outcome variables respectively. All models have adjusted for age, sex (female=0, male=1), education, apolipoprotein E (APOE) ε4 carrier status (non-carrier=0, carrier=1) and clinical diagnosis (MCI=0, AD=1). In addition, when several independent variables were associated with dependent variable, we further estimated the interactive effect between theses variables. In order to the accuracy of this study, we also repeated above analyses by using the WMH volume to replace visual assessment (Fazekas score). Furthermore, we performed same linear regression models in each domain of NPS.

## Results

### Demographic and clinical characteristics

Table 1 summarized the demographic and clinical characteristics of study population. This study included 445 cognitively impaired participants (316 MCI and 129 AD patients). For CSF biomarkers, AD patients had lower CSF Aβ_42_ but higher CSF p-tau_181_, t-tau, p-tau_181_/Aβ_42_, and t-tau/Aβ_42_ than participants with MCI. Moreover, scores of NPI-assessed agitation, disinhibition, motor behavior, delusion, hallucinations, anxiety, apathy, and appetite were higher in AD group versus MCI patients.

**Table 1:** Demographic and clinical data of study population

	Total sample (*N*=445)	MCI (*N*=316)	AD (*N*=129)	*P*
Age	72.53±7.71	71.72±7.26	74.50±8.41	<0.001
Sex (M/F)	250/195	174/142	76/53	0.458
Education	16.18±2.63	16.38±2.61	15.68±2.67	0.012
APOE 4 (carrier/non-carrier)	246/199	160/156	86/43	0.002
CSF biomarkers				
Aβ_42_ (pg/ml)	160.81±50.00	170.40±51.38	137.32±37.30	<0.001
p-tau (pg/ml)	47.59±28.66	25.61±1.44	32.89±2.90	<0.001
t-tau (pg/ml)	99.32±58.71	86.82±52.21	130.50±62.52	<0.001
p-tau/Aβ_42_	0.34±0.25	0.30±0.22	0.45±0.28	<0.001
t-tau/Aβ_42_	0.69±0.53	0.58±0.47	0.96±0.56	<0.001
CSVD markers				
PV-WMH, median (Q1, Q3)	1 (1,2)	1 (1,2)	2 (1,2)	0.012
DM-WMH, median (Q1, Q3)	1 (0,1)	1 (0,1)	1 (0,2)	0.016
WMH volume	7.22±8.98	6.77±8.85	8.33±9.22	0.096
BG-PVS, median (Q1, Q3)	3 (3,3)	3 (3,3)	3 (3,3)	0.780
CS-PVS, median (Q1, Q3)	3 (3,4)	3 (3,4)	3 (3,4)	0.116
Microbleeds, n. (%)	78 (17.5)	46 (14.6)	32 (24.8)	0.009
Lacunes, n. (%)	85 (19.1)	50 (15.8)	35 (27.1)	0.006
NPI				
Hyperactivity	1.79±3.49	1.42±2.98	2.69±4.38	<0.001
Agitation(C)	0.53±1.34	0.42±1.19	0.79±1.63	0.001
Elation(F)	0.07±0.56	0.07±0.48	0.07±0.71	0.652
Disinhibition(H)	0.25±0.94	0.19±0.86	0.38±1.10	0.004
Irritability(I)	0.73±1.69	0.64±1.56	0.95±1.96	0.051
Motor behavior(J)	0.22±0.93	0.10±0.58	0.50±1.43	<0.001
Psychosis	0.99±2.28	0.98±2.23	1.01±2.41	0.832
Delusion(A)	0.12±0.77	0.05±0.43	0.30±1.24	0.002
Hallucinations(B)	0.04±0.33	0.03±0.30	0.07±0.38	0.037
Night-time(K) behavior(K)	0.82±2.23	0.90±2.18	0.64±1.59	0.336
Affective symptoms	1.09±2.00	1.00±2.05	1.30±1.86	0.020
Anxiety(E)	0.49±1.34	0.43±1.36	0.61±1.28	0.012
Depression(D)	0.60±1.18	0.57±1.19	0.68±1.15	0.221
Apathy	1.31±2.90	0.83±2.17	2.48±3.96	<0.001
Apathy(G)	0.77±1.83	0.52±1.54	1.36±2.29	<0.001
Appetite(L)	0.54±1.75	0.31±1.28	1.12±2.47	<0.001
Total	5.17±7.38	4.23±6.52	7.47±8.75	<0.001

*P* values of group comparisons were obtained using the Mann-Whitney U-test and Chi-square test

### Associations of CSF biomarkers and CSVD markers with NPI total score

In the whole sample, the NPI total score was associated on univariate analyses with male (estimate 2.462; 95% CI [1.095, 3.830]; *P* <0.001), education (estimate -0.343; 95% CI [-0.602, -0.084]; *P* =0.010), clinical diagnosis (estimate 3.239; 95% CI [1.753, 4.725]; *P* <0.001), microbleed (estimate 2.977; 95% CI [1.189, 4.765]; *P* =0.001), and lacune (estimate 1.793; 95% CI [0.051, 3.535]; *P* =0.044) (Table 2). Only the association between NPI total scores and microbleed remains significant in univariate (model 2) and multivariable (model 3) analyses after adjusting for age, sex, education, APOE ε4 carrier status, and clinical diagnosis (Table 2).

**Table 2:** Association between NPI total scores with AD biomarkers and CSVD markers in in whole sample

	Model 1^a^		Model 2^b^		Model 3^c^	
	Estimate (95% CI)	*P*	Estimate (95% CI)	*P*	Estimate (95% CI)	*P*
Age	0.042 (-0.047, 0.131)	0.356	-	-	-	-
Sex	2.462 (1.095, 3.830)	**<0.001**	-	-	-	-
Education	-0.343 (-0.602, -0.084)	**0.010**	-	-	-	-
APOE ε4 carrier status	-0.105 (-1.489, 1.279)	0.881	-	-	-	-
Clinical diagnosis	3.239 (1.753, 4.725)	**<0.001**	-	**-**		
Aβ_42_ (pg/ml)	-0.013 (-0.027, 0.001)	0.067	-0.007 (-0.023, 0.008)	0.355	-	-
p-tau_181_ (pg/ml)	0.011 (-0.013, -0.035)	0.388	0.005 (-0.020, 0.030)	0.690	-	-
t-tau (pg/ml)	0.006 (-0.005, 0.018)	0.288	0.003 (-0.010, 0.016)	0.625	-	-
p-tau_181_/Aβ_42_	1.885 (-0.878, 4.648)	0.181	1.103 (-1.862, 4.068)	0.465	-	-
t-tau/Aβ_42_	0.655 (-0.647, 1.958)	0.323	0.295 (-1.141, 1.731)	0.687	-	-
PVL-WMH score	0.147 (-0.677, 0.971)	0.726	-0.023 (-0.951, 0.906)	0.962	-	-
DWM-WMH score	0.182 (-0.617, 0.981)	0.655	0.041 (-0.834, 0.915)	0.927	-	-
BG-PVS score	-0.106 (-1.619, 1.407)	0.891	-0.116 ( -1.586, 1.355)	0.986	-	-
CSO-PVS score	-0.136 (-0.490, 0.218)	0.144	-0.449 (-1.334, 0.436)	0.458	-	-
Microbleeds	2.977 (1.189, 4.765)	**0.001**	2.350 (0.681, 4.019)	**0.006**	2.424 (0.749, 4.099)	**0.005**
Lacune	1.793 (0.051, 3.535)	**0.044**	1.373 (-0.365, 3.112)	0.121	-	-

^a^Model 1 is univariate

^b^Model 2 is univariate (adjusted for age, sex, education, APOE ε4 carrier status, and clinical diagnosis)

^c^Model 3 is step-wise multivariable regression model (adjusted for age, sex, education, APOE ε4 carrier status, and clinical diagnosis)

### Associations of CSF biomarkers and CSVD markers with hyperactivity sub-syndromes

In the whole sample, the hyperactivity sub-syndromes were associated on univariate analyses with male (estimate 1.395; 95% CI [0.752, 2.037]; *P* <0.001), education (estimate -0.154; 95% CI [-0.276, -0.031]; *P* =0.014), clinical diagnosis (estimate 1.269; 95% CI [0.562, 1.976]; *P* <0.001), and microbleed (estimate 1.127; 95% CI [0.278, 1.976]; *P* =0.009) (Table 3). The association between hyperactivity syndrome and microbleed remained significant univariate (model 2) and multivariable (model 3) analyses after adjusting for age, sex, education, APOE ε4 carrier status, and clinical diagnosis (Table 3).

**Table 3:** Association between hyperactivity sub-syndromes with AD biomarkers and CSVD markers in whole sample

	Model 1^a^		Model 2^b^		Model 3^c^	
	Estimate (95% CI)	*P*	Estimate (95% CI)	*P*	Estimate (95% CI)	*P*
Age	0.014 (-0.028, 0.056)	0.508	-	-	-	-
Sex	1.395 (0.752, 2.037)	**<0.001**	-	-	-	-
Education	-0.154 (-0.276, -0.031)	**0.014**	-	-	-	-
APOE ε4 carrier status	-0.164 (-0.818, 0.490)	0.623	-	-	-	-
Clinical diagnosis	1.269 (0.562, 1.976)	**<0.001**	-	-	-	-
Aβ_42_ (pg/ml)	-0.001 (-0.008, 0.005)	0.666	0.002 (-0.006, 0.009)	0.682	-	-
p-tau_181_ (pg/ml)	0.005 (-0.006, 0.017)	0.350	0.005 (-0.007, 0.016)	0.422	-	-
t-tau (pg/ml)	0.001 (-0.004, 0.007)	0.627	0.001 (-0.005, 0.007)	0.716	-	-
p-tau_181_/Aβ_42_	0.800 (-0.507, 2.107)	0.230	0.690 (-0.709, 2.088)	0.333	-	-
t-tau/Aβ_42_	0.153 (-0.463, 0.769)	0.626	0.116 (-0.562, 0.794)	0.737	-	-
PVL-WMH score	0.108 (-0.281, 0.497)	0.586	0.108 (-0.330, 0.546)	0.628	-	-
DWM-WMH score	0.001 (-0.378, 0.378)	1.000	-0.031 (-0.444, 0.382)	0.883	-	-
BG-PVS score	-0.314 (-1.028, 0.401)	0.389	-0.312 (-1.005, 0.382)	0.377	-	-
CSO-PVS score	-0.216 (-0.641, 0.210)	0.320	-0.103 (-0.521, 0.315)	0.628	-	-
Microbleeds	1.127 (0.278, 1.976)	**0.009**	0.934 (0.144, 1.724)	**0.021**	0.925 (0.115, 1.735)	**0.025**
Lacune	0.726 (-0.098, 1.551)	0.084	0.585 (-0.236, 1.406)	0.162	-	-

^a^Model 1 is univariate

^b^Model 2 is univariate (adjusted for age, sex, education, APOE ε4 carrier status, and clinical diagnosis)

^c^Model 3 is step-wise multivariable regression model (adjusted for age, sex, education, APOE ε4 carrier status, and clinical diagnosis)

### Associations of CSF biomarkers and CSVD markers with psychosis sub-syndromes

In the whole sample, the psychosis sub-syndromes did not show significant correlation with AD biomarkers and CSVD markers either in univariate or multivariable analyses (Table 4).

**Table 4:** Association between psychosis sub-syndromes with AD biomarkers and CSVD markers in whole sample

	Model 1^a^		Model 2^b^		Model 3^c^	
	Estimate (95% CI)	*P*	Estimate (95% CI)	*P*	Estimate (95% CI)	*P*
Age	0.013 (-0.014, 0.041)	0.338	-	-	-	-
Sex	-0.061 (-0.490, 0.369)	0.782	-	-	-	-
Education	-0.035 (-0.115, 0.046)	0.400	-	-	-	-
APOE ε4 carrier status	-0.006 (-0.434, 0.422)	0.977	-	-	-	-
Clinical diagnosis	0.030 (-0.439, 0.499)	0.900	-	-	-	-
Aβ_42_ (pg/ml)	-0.001 (-0.005, 0.003)	0.676	-0.001 (-0.006, 0.44)	0.763	-	-
p-tau_181_ (pg/ml)	-0.002 (-0.009, 0.006)	0.670	-0.002 (-0.010, 0.006)	0.629	-	-
t-tau (pg/ml)	0.001 (-0.004, 0.004)	0.979	0.000 (-0.005, 0.004)	0.825	-	-
p-tau_181_/Aβ_42_	-0.141 (-0.998, 0.715)	0.746	-0.200 (-1.158, 0.759)	0.683	-	-
t-tau/Aβ_42_	-0.012 (-0.416, 0.391)	0.952	-0.061 (-0.525, 0.403)	0.795	-	-
PVL-WMH score	0.092 (-0.163, 0.347)	0.479	-0.190 (-0.475, 0.096)	0.192	-	-
DWM-WMH score	0.113 (-0.134, 0.360)	0.369	0.025 (-0.275, 0.325)	0.872	-	-
BG-PVS score	0.059 (-0.409, 0.527)	0.804	0.020 (-0.455, 0.495)	0.935	-	-
CSO-PVS score	-0.155 (-0.433, 0.124)	0.275	-0.190 (-0.474, 0.096)	0.192	-	-
Microbleeds	0.529 (-0.028, 1.087)	0.063	0.440 (-0.102, 0.982)	0.112	-	-
Lacune	0.308 (-0.233, 0.848)	0.264	0.253 (-0.309, 0.816)	0.377	-	-

^a^Model 1 is univariate

^b^Model 2 is univariate (adjusted for age, sex, education, APOE ε4 carrier status, and clinical diagnosis)

^c^Model 3 is step-wise multivariable regression model (adjusted for age, sex, education, APOE ε4 carrier status, and clinical diagnosis)

### Associations of CSF biomarkers and CSVD markers with affective sub-syndromes

The affective symptoms were correlated to CSF Aβ_42_ levels (estimate -0.006; 95% CI [-0.009, -0.002]; *P* =0.003) (Table 5). This association remains unchanged in univariate (model 2) and multivariable (model 3) analyses after adjusting for age, sex, education, APOE ε4 carrier status, and clinical diagnosis (Table 5).

**Table 5:** Association between affective sub-syndromes with AD biomarkers and CSVD markers in whole sample

	Model 1^a^		Model 2^b^		Model 3^c^	
	Estimate (95% CI)	*P*	Estimate (95% CI)	*P*	Estimate (95% CI)	*P*
Age	-0.013 (-0.037, 0.011)	0.301	-	-	-	-
Sex	0.302 (-0.072, 0.676)	0.113	-	-	-	-
Education	-0.039 (-0.110, 0.031)	0.276	-	-	-	-
APOE ε4 carrier status	0.240 (-0.133, 0.614)	0.270	-	-	-	-
Clinical diagnosis	0.291 (-0.118, 0.701)	0.163	-	-	-	-
Aβ_42_ (pg/ml)	-0.006 (-0.009, -0.002)	**0.003**	-0.006 (-0.010, -0.002)	**0.006**	-0.006 (-0.011, -0.002)	**0.005**
p-tau_181_ (pg/ml)	0.004 (-0.003, 0.010)	0.259	0.002 (-0.005, 0.009)	0.499	-	-
t-tau (pg/ml)	0.001 (-0.002, 0.005)	0.359	0.001 (-0.002, 0.005)	0.522	-	-
p-tau_181_/Aβ_42_	0.711 (-0.035, 1.458)	0.062	0.576 (-0.252, 1.405)	0.172	-	-
t-tau/Aβ_42_	0.222 (-0.130, 0.574)	0.216	0.183 (-0.219, 0.585)	0.371	-	-
PVL-WMH score	-0.127 (-0.350, 0.096)	0.263	-0.084 (-0.344, 0.176)	0.526	-	-
DWM-WMH score	-0.003 (-0.260, 0.172)	0.691	0.006 (-0.239, 0.251)	0.962	-	-
BG-PVS score	0.181 (-0.228, 0.590)	0.384	0.228 (-0.183, 0.640)	0.276	-	-
CSO-PVS score	-0.164 (-0.407, 0.080)	0.187	-0.113 (-0.360, 0.135)	0.372	-	-
Microbleeds	0.345 (-0.144, 0.833)	0.167	0.281 (-0.190, 0.751)	0.242	-	-
Lacune	0.139 (-0.335, 0.612)	0.565	0.180 (-0.308, 0.668)	0.468	-	-

^a^Model 1 is univariate

^b^Model 2 is univariate (adjusted for age, sex, education, APOE ε4 carrier status, and clinical diagnosis)

^c^Model 3 is step-wise multivariable regression model (adjusted for age, sex, education, APOE ε4 carrier status, and clinical diagnosis)

### Associations of CSF biomarkers and CSVD markers with apathy sub-syndromes

The apathy sub-syndromes were on univariate analyses associated with male (estimate 0.826; 95% CI [0.286, 1.366]; *P* =0.003), education (estimate -0.115; 95% CI [-0.217, -0.013]; *P* =0.027), clinical diagnosis (estimate 1.648; 95% CI [1.072, 2.225]; *P* <0.001), and microbleed (estimate 0.976; 95% CI [0.270, 1.682]; *P* =0.007) (Table 6). In univariate analysis after adjusting for age, sex, education, APOE ε4 carrier status, and clinical diagnosis, the apathy sub-syndromes showed correlation with CSF t-tau/ Aβ_42_ (estimate 0.372; 95% CI [0.278, 0.618]; *P* =0.044) and microbleed (estimate 0.695; 95% CI [0.041, 1.349]; *P* =0.037) (Table 6). In multivariable regression model 3, the apathy sub-syndromes were related to t-tau/Aβ_42_ (estimate 0.636; 95% CI [0.078, 1.194]; *P* =0.041) and microbleed (estimate 0.693; 95% CI [0.046, 1.340]; *P* =0.036) (Table 6). In addition, we found a significant interactive effect between t-tau/Aβ_42_ and microbleed on apathy sub-syndromes (estimate 0.993; 95% CI [0.360, 1.626]; *P* =0.019) (Supplementary Table 1).

**Table 6:** Association between apathy sub-syndromes with AD biomarkers and CSVD markers in whole sample

	Model 1^a^		Model 2^b^		Model 3^c^	
	Estimate (95% CI)	*P*	Estimate (95% CI)	*P*	Estimate (95% CI)	*P*
Age	0.027 (-0.008, 0.062)	0.132	-	-	-	-
Sex	0.826 (0.286, 1.366)	**0.003**	-	-	-	-
Education	-0.115 (-0.217, -0.013)	**0.027**	-	-	-	-
APOE ε4 carrier status	-0.175 (-0.719, 0.369)	0.527	-	-	-	-
Clinical diagnosis	1.648 (1.072, 2.225)	**<0.001**	-	-		
Aβ_42_ (pg/ml)	-0.005 (-0.010, 0.001)	0.082	-0.002 (-0.008, 0.004)	0.509	-	-
p-tau_181_ (pg/ml)	0.003 (-0.006, -0.012)	0.527	0.000 (-0.010, 0.010)	0.974	-	-
t-tau (pg/ml)	0.003 (-0.001, 0.008)	0.132	0.001 (-0.004, 0.006)	0.596	-	-
p-tau_181_/Aβ_42_	0.515 (-0.573, 1.603)	0.353	0.037 (-1.121, 1.195)	0.950	-	-
t-tau/Aβ_42_	0.293 (-0.220, 0.805)	0.262	0.372 (0.278, 0.618)	**0.044**	0.636 (0.078, 1.194)	**0.041**
PVL-WMH score	0.074 (-0.250, 0.398)	0.653	-0.071 (-0.434, 0.291)	0.699	-	-
DWM-WMH score	0.113 (-0.202, 0.427)	0.482	0.008 (-0.333, 0.350)	0.962	-	-
BG-PVS score	-0.032 (-0.627, 0.563)	0.915	-0.052 (-0.626, 0.522)	0.860	-	-
CSO-PVS score	-0.136 (-0.490, 0.218)	0.452	-0.043 (-0.389, 0.302)	0.805	-	-
Microbleeds	0.976 (0.270, 1.682)	**0.007**	0.695 (0.041, 1.349)	**0.037**	0.693 (0.046, 1.340)	**0.036**
Lacune	0.620 (-0.066, 1.306)	0.076	0.522 (-0.175, 1.218)	0.142	-	-

^a^Model 1 is univariate

^b^Model 2 is univariate (adjusted for age, sex, education, APOE ε4 carrier status, and clinical diagnosis)

^c^Model 3 is step-wise multivariable regression model (adjusted for age, sex, education, APOE ε4 carrier status, and clinical diagnosis)

### Additional analyses

When including the total WMH volume instead of Fazekas score, the results remain unchanged (Supplementary Table 2-6). In addition, the results of linear regression models in each NPS domain were showed in Supplementary Table 7-18.

## Discussion

In this study, we assessed the relationships of neuropsychiatric sub-syndromes, AD-related pathology, and SVD markers in cognitively impaired patients. Our findings are consistent with our initial hypothesis that AD pathology and vascular damage not only contribute to various psychiatric symptoms but also attack on same sub-syndrome synergistically. We found that: 1) CSF Aβ_42_ was related to affective sub-syndrome; 2) microbleed was associated with hyperactivity and NPI total scores; 3) both microbleed and CSF t-tau/Aβ_42_ were associated with apathetic symptom.

We found a negative association between CSF Aβ_42_ and affective symptoms including anxiety and depression. As previously demonstrated, lower CSF Aβ_42_ was related to more amyloid deposition, which has been found to be associated with more severe anxiety and depressive symptoms [9, 28, 29]. Two studies from the Netherlands have reported that the presence of anxiety was associated with abnormal CSF Aβ_42_, p-tau, and t-tau in individuals across the AD spectrum [29, 30]. Similarly, Krell-Roesch et al. observed that lower levels of CSF Aβ_42_ correlated with severer depressive and anxiety symptoms in cognitively unimpaired individuals [10]. As a core pathologic change of AD, amyloid deposition may drive the psychiatric symptoms [10]. In animal models, researchers have observed that amyloid plaque could cause the deficits of neurotransmitter systems, which is related to behavioral abnormalities [31]. Also, it has been reported that higher Aβ burden was associated with increasing anxious and depressive symptoms in both cognitively normal and cognitive impaired individuals [11, 32]. Therefore, affective subsyndrome may be mainly affected by amyloid deposition.

Furthermore, we also found the association between microbleed with NPI total scores and hyperactive sub-syndrome. Microbleed is a kind of imaging markers of cerebral SVD, which has been found that it could aggravate some psychiatric symptoms [33, 34]. In a community-based Asian cohort, Xu et al. observed that the presence of microbleed was associated with apathy, disinhibition and depression [33]. Also, previous study has found the relationship between SVD burden and multiple NPS, suggesting the driven role of vascular pathology to psychiatric symptoms[15]. The potential mechanisms may be that SVD could result in the degeneration of neurons and neural disconnections [35, 36], which further lead to disruption of functional network associated with NPS [37, 38].

Notably, we observed that apathy was associated with both microbleed and CSF t-tau/Aβ_42_. Apathy is the most common psychiatric symptoms in cognitively impaired patients[39]. Previously, researchers have found that apathic symptom was associated with amyloidosis and tauopathy based on CSF biomarkers or PET technique in cognitively impaired individuals [11, 40]. It has been observed that apathy correlated with prefrontal Aβ deposition in AD and MCI patients [41, 42]. In AD animal models, researchers have found that Aβ oligomers could reduce the glutamatergic synaptic transmission strength and plasticity, which further influence the neuronal activity [43]. Tau pathology may also result in focal neurotoxicity and the network disruption, leading to the appearance of apathy [40]. Moreover, previous studies have reported that increased severity of microbleed is associated with more serious apathy symptom [15, 44]. Thus, we considered that AD pathology and microbleed have synergistical effects on disruption of several cerebral networks which are related to the emergence of apathy. Methylphenidate may treat apathy symptoms by enhancing norepinephrine and dopamine actions in related neural circuits disrupted by AD and SVD pathology [45].

In addition, in our study we only observed the associations between hyperactive and apathic syndromes with microbleed but not WMH. One potential explanation is that our study population consisted of cognitive impaired patients rather than non-demented individuals [15]. Also, the mechanisms of WMH and microbleed could be various. Previous study has described the relationship between microbleed and amyloid [46]. Therefore, the microbleeds might play more central role in NPS than other SVD markers.

There are still several limitations in this study. First, the causal direction of the association among AD pathology, SVD, and NPS in this study cannot be established due to the cross-sectional study design. However, growing evidence implicates the psychiatric symptoms as consequences of AD pathology and vascular change [10, 14]. Thus, we considered the NPS as the outcome in statistical model to estimate the effect of AD pathology and SVD on NPS. Second, in the present study, we assessed the severity of NPS only by the NPI rather than more specific test such as Beck Depression Inventory II (BDI-II) and Beck Anxiety Inventory (BAI). Further studies should apply more accurate assessment for disentangling the relationship between the pathological burden and individual psychiatric symptom scores. Third, the sample size is relatively small, and the number of MCI patients in the study population was more than AD patients so that the NPS burdens were relatively lower. Studies with large sample size is needed to further confirmed the association of NPS, SVD and AD pathology especially in different clinical stage.

The strength of our study is to disentangling the distinct effects of AD biomarkers and SVD markers on neuropsychiatric subsyndromes in cognitively impaired individuals. Consist with previous studies, we found that CSF Aβ_42_ and microbleed were associated with affective and hyperactive sub-syndrome respectively. A novel finding is that we detected significant interaction between microbleed and CSF t-tau/Aβ_42_ on apathy subsyndrome, indicating the synergistic effect of AD pathology and vascular damage to some extent. Thus, we considered that AD pathology and vascular damage could influence the common and different NPS-related neural pathway, which further lead to the appearance of specific symptoms.

## Conclusion

Our study among cognitively impaired patients showed that CSF Aβ_42_ was associated with affective symptoms, and microbleed was correlated with hyperactivity and apathy, suggesting the effect of AD pathology and SVD on different neuropsychiatric sub-syndromes. This should be taken into account in further research focusing on therapy of NPS.

### Supplementary Information

**Supplementary Material 1.** 

## Data Availability

The data used in the preparation of this article were obtained from the Alzheimer’s disease Neuroimaging Initiative (ADNI) database: http://adni.loni.usc.edu/.

## Abbreviations

NPS: Neuropsychiatric symptoms
AD: Alzheimer’s disease
MCI: Mild cognitive impairment
CSF: Cerebrospinal fluid
SVD: Small vessel disease
WMS-R: Wechsler Memory Scale-Revised
CDR: Clinical Dementia Rating
MMSE: Mini-Mental State Examination
NPI: Neuropsychiatric Inventory
MoCA: Montreal Cognitive Assessment
AVLT: Auditory Verbal Learning Test
CDT: Clock Drawing Test
TMT-A: Trial-Making Test-A
TMT-B: Trial-Making Test-B
BNT: Boston Naming Test
Aβ: Amyloid-beta
p-tau_181_: Tau phosphorylated at threonine 181
t-tau: Total tau
WMH: White matter hyperintensities
EPVS: Enlarged perivascular spaces
BG: Basal ganglia
CSO: Centrum semiovale
