# Supplementary material for: Cerebrospinal fluid amyloid-β and cerebral microbleed are associated with distinct neuropsychiatric sub-syndromes in cognitively impaired patients
Source: Alzheimers Res Ther. 2024 Apr 3;16:69. doi: 10.1186/s13195-024-01434-7 (PMC10988961; doi:10.1186/s13195-024-01434-7)
Supplement: Supplementary file 1 — Supplementary Material 1. [file 13195_2024_1434_MOESM1_ESM.docx]

**Supplementary Table 1. Linear regression model for investigating the interactive effect between AD pathology and SVD on apathy sub-syndromes in whole sample.**

|  | Model 1^a^ | | Model 2^b^ | | Model 3^c^ | |
| --- | --- | --- | --- | --- | --- | --- |
|  | Estimate (95% CI) | *P* | Estimate (95% CI) | *P* | Estimate (95% CI) | *P* |
| Age | 0.027 (-0.008, 0.062) | 0.132 | - | - | - | - |
| Sex | 0.826 (0.286, 1.366) | **0.003** | - | - | - | - |
| Education | -0.115 (-0.217, -0.013) | **0.027** | - | - | - | - |
| APOE ε4 carrier status | -0.175 (-0.719, 0.369) | 0.527 | - | - | - | - |
| Clinical diagnosis | 1.648 (1.072, 2.225) | **<0.001** | - | - | - | - |
| Aβ_42_ (pg/ml) | -0.005 (-0.010, 0.001) | 0.082 | -0.002 (-0.008, 0.004) | 0.509 | - | - |
| p-tau_181_ (pg/ml) | 0.003 (-0.006, -0.012) | 0.527 | 0.000 (-0.010, 0.010) | 0.974 | - | - |
| t-tau (pg/ml) | 0.003 (-0.001, 0.008) | 0.132 | 0.001 (-0.004, 0.006) | 0.596 | - | - |
| p-tau_181_/Aβ_42_ | 0.515 (-0.573, 1.603) | 0.353 | 0.037 (-1.121, 1.195) | 0.950 | - | - |
| t-tau/Aβ_42_ | 0.293 (-0.220, 0.805) | 0.262 | 0.372 (0.278, 0.618) | **0.044** | - | - |
| PVL-WMH score | 0.074 (-0.250, 0.398) | 0.653 | -0.071 (-0.434, 0.291) | 0.699 | - | - |
| DWM-WMH score | 0.113 (-0.202, 0.427) | 0.482 | 0.008 (-0.333, 0.350) | 0.962 | - | - |
| BG-PVS score | -0.032 (-0.627, 0.563) | 0.915 | -0.052 (-0.626, 0.522) | 0.860 | - | - |
| CSO-PVS score | -0.136 (-0.490, 0.218) | 0.452 | -0.043 (-0.389, 0.302) | 0.805 | - | - |
| Microbleeds | 0.976 (0.270, 1.682) | **0.007** | 0.695 (0.041, 1.349) | **0.037** | - | - |
| Lacune | 0.620 (-0.066, 1.306) | 0.076 | 0.522 (-0.175, 1.218) | 0.142 | - | - |
| Microbleed×t-tau/Aβ_42_ | 0.692 (0.054, 1.330) | **0.011** | 0.692 (0.054, 1.330) | **0.034** | 0.993 (0.360, 1.626) | **0.019** |

^a^ Model 1 is univariate.

^b^ Model 2 is univariate (adjusted for age, sex, education, APOE ε4 carrier status, and clinical diagnosis).

^c^ Model 3 is step-wise multivariable regression model (adjusted for age, sex, education, APOE ε4 carrier status, and clinical diagnosis).

**Supplementary Table 2. Association between NPI total score with AD biomarkers and CSVD markers in whole sample.**

|  | Model 1^a^ | | Model 2^b^ | | Model 3^c^ | |
| --- | --- | --- | --- | --- | --- | --- |
|  | Estimate (95% CI) | *P* | Estimate (95% CI) | *P* | Estimate (95% CI) | *P* |
| Age | 0.042 (-0.047, 0.131) | 0.356 | - | - | - | - |
| Sex | 2.462 (1.095, 3.830) | **<0.001** | - | - | - | - |
| Education | -0.343 (-0.602, -0.084) | **0.010** | - | - | - | - |
| APOE ε4 carrier status | -0.105 (-1.489, 1.279) | 0.881 | - | - | - | - |
| Clinical diagnosis | 3.239 (1.753, 4.725) | **<0.001** | - | - | - | - |
| Aβ_42_ (pg/ml) | -0.013 (-0.027, 0.001) | 0.067 | -0.007 (-0.023, 0.008) | 0.355 | - | - |
| p-tau_181_ (pg/ml) | 0.011 (-0.013, -0.035) | 0.388 | 0.005 (-0.020, 0.030) | 0.690 | - | - |
| t-tau (pg/ml) | 0.006 (-0.005, 0.018) | 0.288 | 0.003 (-0.010, 0.016) | 0.625 | - | - |
| p-tau_181_/Aβ_42_ | 1.885 (-0.878, 4.648) | 0.181 | 1.103 (-1.862, 4.068) | 0.465 | - | - |
| t-tau/Aβ_42_ | 0.655 (-0.647, 1.958) | 0.323 | 0.295 (-1.141, 1.731) | 0.687 | - | - |
| WMH volume | 0.054 (-0.023, 0.130) | 0.169 | 0.024 (-0.056, 0.103) | 0.558 |  |  |
| BG-PVS score | -0.106 (-1.619, 1.407) | 0.891 | -0.116 ( -1.586, 1.355) | 0.986 | - | - |
| CSO-PVS score | -0.136 (-0.490, 0.218) | 0.144 | -0.449 (-1.334, 0.436) | 0.458 | - | - |
| Microbleeds | 2.977 (1.189, 4.765) | **0.001** | 2.350 (0.681, 4.019) | **0.006** | 2.905 (1.145, 4.665) | **0.001** |
| Lacune | 1.793 (0.051, 3.535) | **0.044** | 1.373 (-0.365, 3.112) | 0.121 | - | - |

^a^ Model 1 is univariate.

^b^ Model 2 is univariate (adjusted for age, sex, education, APOE ε4 carrier status, and clinical diagnosis).

^c^ Model 3 is step-wise multivariable regression model (adjusted for age, sex, education, APOE ε4 carrier status, and clinical diagnosis).

**Supplementary Table 3. Association between hyperactivity sub-syndromes with AD biomarkers and CSVD markers in whole sample.**

|  | Model 1^a^ | | Model 2^b^ | | Model 3^c^ | |
| --- | --- | --- | --- | --- | --- | --- |
|  | Estimate (95% CI) | *P* | Estimate (95% CI) | *P* | Estimate (95% CI) | *P* |
| Age | 0.014 (-0.028, 0.056) | 0.508 | - | - | - | - |
| Sex | 1.395 (0.752, 2.037) | **<0.001** | - | - | - | - |
| Education | -0.154 (-0.276, -0.031) | **0.014** | - | - | - | - |
| APOE ε4 carrier status | -0.164 (-0.818, 0.490) | 0.623 | - | - | - | - |
| Clinical diagnosis | 1.269 (0.562, 1.976) | **<0.001** | - | - | - | - |
| Aβ_42_ (pg/ml) | -0.001 (-0.008, 0.005) | 0.666 | 0.002 (-0.006, 0.009) | 0.682 | - | - |
| p-tau_181_ (pg/ml) | 0.005 (-0.006, 0.017) | 0.350 | 0.005 (-0.007, 0.016) | 0.422 | - | - |
| t-tau (pg/ml) | 0.001 (-0.004, 0.007) | 0.627 | 0.001 (-0.005, 0.007) | 0.716 | - | - |
| p-tau_181_/Aβ_42_ | 0.800 (-0.507, 2.107) | 0.230 | 0.690 (-0.709, 2.088) | 0.333 | - | - |
| t-tau/Aβ_42_ | 0.153 (-0.463, 0.769) | 0.626 | 0.116 (-0.562, 0.794) | 0.737 | - | - |
| WMH volume | 0.022 (-0.014, 0.058) | 0.236 | 0.008 (-0.029, 0.046) | 0.661 |  |  |
| BG-PVS score | -0.314 (-1.028, 0.401) | 0.389 | -0.312 (-1.005, 0.382) | 0.377 | - | - |
| CSO-PVS score | -0.216 (-0.641, 0.210) | 0.320 | -0.103 (-0.521, 0.315) | 0.628 | - | - |
| Microbleeds | 1.127 (0.278, 1.976) | **0.009** | 0.934 (0.144, 1.724) | **0.021** | 1.104 (0.252, 1.956) | **0.011** |
| Lacune | 0.726 (-0.098, 1.551) | 0.084 | 0.585 (-0.236, 1.406) | 0.162 | - | - |

^a^ Model 1 is univariate.

^b^ Model 2 is univariate (adjusted for age, sex, education, APOE ε4 carrier status, and clinical diagnosis).

^c^ Model 3 is step-wise multivariable regression model (adjusted for age, sex, education, APOE ε4 carrier status, and clinical diagnosis).

**Supplementary Table 4. Association between psychosis sub-syndromes with AD biomarkers and CSVD markers in whole sample.**

|  | Model 1^a^ | | Model 2^b^ | | Model 3^c^ | |
| --- | --- | --- | --- | --- | --- | --- |
|  | Estimate (95% CI) | *P* | Estimate (95% CI) | *P* | Estimate (95% CI) | *P* |
| Age | 0.013 (-0.014, 0.041) | 0.338 | - | - | - | - |
| Sex | -0.061 (-0.490, 0.369) | 0.782 | - | - | - | - |
| Education | -0.035 (-0.115, 0.046) | 0.400 | - | - | - | - |
| APOE ε4 carrier status | -0.006 (-0.434, 0.422) | 0.977 | - | - | - | - |
| Clinical diagnosis | 0.030 (-0.439, 0.499) | 0.900 | - | - | - | - |
| Aβ_42_ (pg/ml) | -0.001 (-0.005, 0.003) | 0.676 | -0.001 (-0.006, 0.44) | 0.763 | - | - |
| p-tau_181_ (pg/ml) | -0.002 (-0.009, 0.006) | 0.670 | -0.002 (-0.010, 0.006) | 0.629 | - | - |
| t-tau (pg/ml) | 0.001 (-0.004, 0.004) | 0.979 | 0.000 (-0.005, 0.004) | 0.825 | - | - |
| p-tau_181_/Aβ_42_ | -0.141 (-0.998, 0.715) | 0.746 | -0.200 (-1.158, 0.759) | 0.683 | - | - |
| t-tau/Aβ_42_ | -0.012 (-0.416, 0.391) | 0.952 | -0.061 (-0.525, 0.403) | 0.795 | - | - |
| WMH volume | 0.009 (-0.015, 0.033) | 0.466 | 0.005 (-0.021, 0.031) | 0.712 | - | - |
| BG-PVS score | 0.059 (-0.409, 0.527) | 0.804 | 0.020 (-0.455, 0.495) | 0.935 | - | - |
| CSO-PVS score | -0.155 (-0.433, 0.124) | 0.275 | -0.190 (-0.474, 0.096) | 0.192 | - | - |
| Microbleeds | 0.529 (-0.028, 1.087) | 0.063 | 0.440 (-0.102, 0.982) | 0.112 | - | - |
| Lacune | 0.308 (-0.233, 0.848) | 0.264 | 0.253 (-0.309, 0.816) | 0.377 | - | - |

^a^ Model 1 is univariate.

^b^ Model 2 is univariate (adjusted for age, sex, education, APOE ε4 carrier status, and clinical diagnosis).

^c^ Model 3 is step-wise multivariable regression model (adjusted for age, sex, education, APOE ε4 carrier status, and clinical diagnosis).

**Supplementary Table 5. Association between affective sub-syndromes with AD biomarkers and CSVD markers in whole sample.**

|  | Model 1^a^ | | Model 2^b^ | | Model 3^c^ | |
| --- | --- | --- | --- | --- | --- | --- |
|  | Estimate (95% CI) | *P* | Estimate (95% CI) | *P* | Estimate (95% CI) | *P* |
| Age | -0.013 (-0.037, 0.011) | 0.301 | - | - | - | - |
| Sex | 0.302 (-0.072, 0.676) | 0.113 | - | - | - | - |
| Education | -0.039 (-0.110, 0.031) | 0.276 | - | - | - | - |
| APOE ε4 carrier status | 0.240 (-0.133, 0.614) | 0.270 | - | - | - | - |
| Clinical diagnosis | 0.291 (-0.118, 0.701) | 0.163 | - | - | - | - |
| Aβ_42_ (pg/ml) | -0.006 (-0.009, -0.002) | **0.003** | -0.006 (-0.010, -0.002) | **0.006** | -0.006 (-0.011, -0.002) | **0.005** |
| p-tau_181_ (pg/ml) | 0.004 (-0.003, 0.010) | 0.259 | 0.002 (-0.005, 0.009) | 0.499 | - | - |
| t-tau (pg/ml) | 0.001 (-0.002, 0.005) | 0.359 | 0.001 (-0.002, 0.005) | 0.522 | - | - |
| p-tau_181_/Aβ_42_ | 0.711 (-0.035, 1.458) | 0.062 | 0.576 (-0.252, 1.405) | 0.172 | - | - |
| t-tau/Aβ_42_ | 0.222 (-0.130, 0.574) | 0.216 | 0.183 (-0.219, 0.585) | 0.371 | - | - |
| WMH volume | 0.005 (-0.016, 0.026) | 0.635 | 0.007 (-0.015, 0.029) | 0.542 | - | - |
| BG-PVS score | 0.181 (-0.228, 0.590) | 0.384 | 0.228 (-0.183, 0.640) | 0.276 | - | - |
| CSO-PVS score | -0.164 (-0.407, 0.080) | 0.187 | -0.113 (-0.360, 0.135) | 0.372 | - | - |
| Microbleeds | 0.345 (-0.144, 0.833) | 0.167 | 0.281 (-0.190, 0.751) | 0.242 | - | - |
| Lacune | 0.139 (-0.335, 0.612) | 0.565 | 0.180 (-0.308, 0.668) | 0.468 | - | - |

^a^ Model 1 is univariate.

^b^ Model 2 is univariate (adjusted for age, sex, education, APOE ε4 carrier status, and clinical diagnosis).

^c^ Model 3 is step-wise multivariable regression model (adjusted for age, sex, education, APOE ε4 carrier status, and clinical diagnosis).

**Supplementary Table 6. Association between apathy sub-syndromes with AD biomarkers and CSVD markers in whole sample.**

|  | Model 1^a^ | | Model 2^b^ | | Model 3^c^ | |
| --- | --- | --- | --- | --- | --- | --- |
|  | Estimate (95% CI) | *P* | Estimate (95% CI) | *P* | Estimate (95% CI) | *P* |
| Age | 0.027 (-0.008, 0.062) | 0.132 | - | - | - | - |
| Sex | 0.826 (0.286, 1.366) | **0.003** | - | - | - | - |
| Education | -0.115 (-0.217, -0.013) | **0.027** | - | - | - | - |
| APOE ε4 carrier status | -0.175 (-0.719, 0.369) | 0.527 | - | - | - | - |
| Clinical diagnosis | 1.648 (1.072, 2.225) | **<0.001** | - | - | - | - |
| Aβ_42_ (pg/ml) | -0.005 (-0.010, 0.001) | 0.082 | -0.002 (-0.008, 0.004) | 0.509 | - | - |
| p-tau_181_ (pg/ml) | 0.003 (-0.006, -0.012) | 0.527 | 0.000 (-0.010, 0.010) | 0.974 | - | - |
| t-tau (pg/ml) | 0.003 (-0.001, 0.008) | 0.132 | 0.001 (-0.004, 0.006) | 0.596 | - | - |
| p-tau_181_/Aβ_42_ | 0.515 (-0.573, 1.603) | 0.353 | 0.037 (-1.121, 1.195) | 0.950 | - | - |
| t-tau/Aβ_42_ | 0.293 (-0.220, 0.805) | 0.262 | 0.372 (0.278, 0.618) | **0.044** | 0.653 (0.122, 1.207) | **0.023** |
| WMH volume | 0.018 (-0.012, 0.048) | 0.242 | 0.004 (-0.028, 0.035) | 0.822 | - | - |
| BG-PVS score | -0.032 (-0.627, 0.563) | 0.915 | -0.052 (-0.626, 0.522) | 0.860 | - | - |
| CSO-PVS score | -0.136 (-0.490, 0.218) | 0.452 | -0.043 (-0.389, 0.302) | 0.805 | - | - |
| Microbleeds | 0.976 (0.270, 1.682) | **0.007** | 0.695 (0.041, 1.349) | **0.037** | 0.839 (0.159, 1.520) | **0.016** |
| Lacune | 0.620 (-0.066, 1.306) | 0.076 | 0.522 (-0.175, 1.218) | 0.142 | - | - |

^a^ Model 1 is univariate.

^b^ Model 2 is univariate (adjusted for age, sex, education, APOE ε4 carrier status, and clinical diagnosis).

^c^ Model 3 is step-wise multivariable regression model (adjusted for age, sex, education, APOE ε4 carrier status, and clinical diagnosis).

**Supplementary Table 7. Association between** **delusion with AD biomarkers and CSVD markers in whole sample. [A]**

|  | Model 1^a^ | | Model 2^b^ | | Model 3^c^ | |
| --- | --- | --- | --- | --- | --- | --- |
|  | Estimate (95% CI) | *P* | Estimate (95% CI) | *P* | Estimate (95% CI) | *P* |
| Age | 0.011 (0.002, 0.020) | **0.022** | - | - | - | - |
| Sex | -0.045 (-0.189, 0.099) | 0.542 | - | - | - | - |
| Education | -0.011 (-0.038, 0.016) | 0.427 | - | - | - | - |
| APOE ε4 carrier status | -0.013 (-0.156, 0.131) | 0.861 | - | - | - | - |
| Clinical diagnosis | 0.252 (0.096, 0.407) | **0.002** | - | - | - | - |
| Aβ_42_ (pg/ml) | -0.001 (-0.003, 0.000) | 0.092 | -0.001 (-0.002, 0.001) | 0.376 | - | - |
| p-tau_181_ (pg/ml) | 0.001 (-0.002, 0.003) | 0.606 | -0.001 (-0.003, 0.003) | 0.941 | - | - |
| t-tau (pg/ml) | 0.001 (-0.001, 0.002) | 0.344 | 0.000 (-0.002, 0.001) | 0.839 | - | - |
| p-tau_181_/Aβ_42_ | 0.115 (-0.172, 0.402) | 0.431 | 0.010 (-0.306, 0.327) | 0.950 | - | - |
| t-tau/Aβ_42_ | 0.070 (-0.065, 0.206) | 0.306 | -0.007 (-0.161, 0.146) | 0.935 | - | - |
| PVL-WMH score | 0.106 (0.021, 0.191) | **0.021** | 0.062 (-0.037, 0.161) | 0.216 | - | - |
| DWM-WMH score | 0.110 (0.028, 0.193) | **0.009** | 0.074 (-0.019, 0.167) | 0.117 | - | - |
| BG-PVS score | 0.078 (-0.079, 0.235) | 0.328 | 0.060 (-0.097, 0.217) | 0.456 | - | - |
| CSO-PVS score | 0.067 (-0.026, 0.160) | 0.160 | 0.064 (-0.030, 0.158) | 0.184 | - | - |
| Microbleeds | 0.099 (-0.078, 0.277) | 0.273 | 0.065 (-0.124, 0.253) | 0.503 | - | - |
| Lacune | 0.240 (0.060, 0.420) | **0.009** | 0.174 (-0.012, 0.359) | 0.066 | - | - |

^a^ Model 1 is univariate.

^b^ Model 2 is univariate (adjusted for age, sex, education, APOE ε4 carrier status, and clinical diagnosis).

^c^ Model 3 is step-wise multivariable regression model (adjusted for age, sex, education, APOE ε4 carrier status, and clinical diagnosis).

**Supplementary Table 8. Association between hallucinations with AD biomarkers and CSVD markers in whole sample. [B]**

|  | Model 1^a^ | | Model 2^b^ | | Model 3^c^ | |
| --- | --- | --- | --- | --- | --- | --- |
|  | Estimate (95% CI) | *P* | Estimate (95% CI) | *P* | Estimate (95% CI) | *P* |
| Age | 0.002 (-0.002, 0.006) | 0.308 | - | - | - | - |
| Sex | -0.019 (-0.081, 0.042) | 0.537 | - | - | - | - |
| Education | 0.009 (-0.003, 0.020) | 0.140 | - | - | - | - |
| APOE ε4 carrier status | 0.055 (-0.006, 0.116) | 0.077 | - | - | - | - |
| Clinical diagnosis | 0.041 (-0.026, 0.108) | 0.226 | - | - | - | - |
| Aβ_42_ (pg/ml) | -0.001 (-0.001, 0.000) | 0.056 | 0.000 (-0.001, 0.000) | 0.391 | - | - |
| p-tau_181_ (pg/ml) | 0.001 (0.000, 0.002) | 0.200 | 0.000 (-0.001, 0.002) | 0.475 | - | - |
| t-tau (pg/ml) | 0.000 (0.000, 0.001) | 0.541 | 0.000 (-0.001, 0.000) | 0.718 | - | - |
| p-tau_181_/Aβ_42_ | 0.114 (-0.008, 0.236) | 0.068 | 0.074 (-0.061, 0.210) | 0.282 | - | - |
| t-tau/Aβ_42_ | 0.040 (-0.018, 0.098) | 0.173 | 0.012 (-0.054, 0.078) | 0.716 | - | - |
| PVL-WMH score | 0.018 (-0.019, 0.054) | 0.345 | 0.010 (-0.032, 0.053) | 0.641 | - | - |
| DWM-WMH score | -0.012 (-0.047, 0.024) | 0.511 | -0.027 (-0.067, 0.013) | 0.182 | - | - |
| BG-PVS score | 0.018 (-0.048, 0.085) | 0.588 | 0.022 (-0.046, 0.089) | 0.526 | - | - |
| CSO-PVS score | -0.006 (-0.046, 0.034) | 0.775 | -0.008 (-0.048, 0.033) | 0.704 | - | - |
| Microbleeds | 0.011 (-0.065, 0.087) | 0.782 | -0.006 (-0.087, 0.075) | 0.883 | - | - |
| Lacune | 0.052 (-0.025, 0.129) | 0.188 | 0.043 (-0.037, 0.122) | 0.291 | - | - |

^a^ Model 1 is univariate.

^b^ Model 2 is univariate (adjusted for age, sex, education, APOE ε4 carrier status, and clinical diagnosis).

^c^ Model 3 is step-wise multivariable regression model (adjusted for age, sex, education, APOE ε4 carrier status, and clinical diagnosis).

**Supplementary Table 9. Association between agitation with AD biomarkers and CSVD markers in whole sample. [C]**

|  | Model 1^a^ | | Model 2^b^ | | Model 3^c^ | |
| --- | --- | --- | --- | --- | --- | --- |
|  | Estimate (95% CI) | *P* | Estimate (95% CI) | *P* | Estimate (95% CI) | *P* |
| Age | 0.000 (-0.016, 0.017) | 0.965 | - | - | - | - |
| Sex | 0.516 (0.269, 0.763) | **<0.001** | - | - | - | - |
| Education | -0.032 (-0.079, 0.015) | 0.183 | - | - | - | - |
| APOE ε4 carrier status | 0.033 (-0.218, 0.284) | 0.796 | - | - | - | - |
| Clinical diagnosis | 0.373 (0.100, 0.646) | **0.007** | - | - | - | - |
| Aβ_42_ (pg/ml) | 0.000 (-0.002, 0.003) | 0.713 | 0.002 (-0.001, 0.005) | 0.147 | - | - |
| p-tau_181_ (pg/ml) | 0.001 (-0.003, 0.005) | 0.666 | 0.001 (-0.004, 0.005) | 0.824 | - | - |
| t-tau (pg/ml) | 0.000 (-0.002, 0.002) | 0.858 | 0.000 (-0.003, 0.002) | 0.713 | - | - |
| p-tau_181_/Aβ_42_ | 0.145 (-0.357, 0.647) | 0.570 | 0.050 (-0.495, 0.594) | 0.858 | - | - |
| t-tau/Aβ_42_ | 0.015 (-0.221, 0.252) | 0.900 | -0.008 (-0.272, 0.256) | 0.954 | - | - |
| PVL-WMH score | -0.104 (-0.253, 0.045) | 0.170 | -0.118 (-0.288, 0.052) | 0.175 | - | - |
| DWM-WMH score | -0.075 (-0.220, 0.070) | 0.311 | -0.075 (-0.235, 0.086) | 0.360 | - | - |
| BG-PVS score | -0.043 (-0.317, 0.232) | 0.761 | -0.019 (-0.289, 0.251) | 0.891 | - | - |
| CSO-PVS score | -0.037 (-0.200, 0.126) | 0.656 | 0.010 (-0.153, 0.173) | 0.905 | - | - |
| Microbleeds | 0.069 (-0.242, 0.380) | 0.664 | 0.079 (-0.246, 0.405) | 0.631 | - | - |
| Lacune | -0.010 (-0.328, 0.307) | 0.950 | -0.042 (-0.362, 0.278) | 0.795 | - | - |

^a^ Model 1 is univariate.

^b^ Model 2 is univariate (adjusted for age, sex, education, APOE ε4 carrier status, and clinical diagnosis).

^c^ Model 3 is step-wise multivariable regression model (adjusted for age, sex, education, APOE ε4 carrier status, and clinical diagnosis).

**Supplementary Table 10. Association between depression with AD biomarkers and CSVD markers in whole sample. [D]**

|  | Model 1^a^ | | Model 2^b^ | | Model 3^c^ | |
| --- | --- | --- | --- | --- | --- | --- |
|  | Estimate (95% CI) | *P* | Estimate (95% CI) | *P* | Estimate (95% CI) | *P* |
| Age | -0.001 (-0.015, 0.013) | 0.890 | - | - | - | - |
| Sex | 0.150 (-0.072, 0.372) | 0.184 | - | - | - | - |
| Education | 0.004 (-0.037, 0.046) | 0.834 | - | - | - | - |
| APOE ε4 carrier status | 0.080 (-0.141, 0.302) | 0.476 | - | - | - | - |
| Clinical diagnosis | 0.113 (-0.130, 0.355) | 0.363 | - | - | - | - |
| Aβ_42_ (pg/ml) | -0.002 (-0.005, 0.000) | **0.036** | -0.002 (-0.005, 0.000) | 0.063 | - | - |
| p-tau_181_ (pg/ml) | 0.000 (-0.003, 0.004) | 0.808 | 0.000 (-0.004, 0.004) | 0.974 | - | - |
| t-tau (pg/ml) | 0.000 (-0.001, 0.002) | 0.638 | 0.001 (-0.002, 0.003) | 0.624 | - | - |
| p-tau_181_/Aβ_42_ | 0.209 (-0.234, 0.653) | 0.354 | 0.166 (-0.329, 0.662) | 0.509 | - | - |
| t-tau/Aβ_42_ | 0.061 (-0.148, 0.270) | 0.565 | 0.047 (-0.193, 0.287) | 0.701 | - | - |
| PVL-WMH score | -0.005 (-0.137, 0.127) | 0.938 | 0.011 (-0.145, 0.166) | 0.893 | - | - |
| DWM-WMH score | -0.017 (-0.145, 0.111) | 0.792 | -0.008 (-0.154, 0.138) | 0.919 | - | - |
| BG-PVS score | 0.058 (-0.185, 0.300) | 0.641 | 0.077 (-0.168, 0.323) | 0.536 | - | - |
| CSO-PVS score | -0.059 (-0.203, 0.085) | 0.421 | -0.045 (-0.193, 0.103) | 0.552 | - | - |
| Microbleeds | 0.137 (-0.137, 0.412) | 0.327 | 0.163 (-0.133, 0.458) | 0.280 | - | - |
| Lacune | 0.186 (-0.094, 0.466) | 0.192 | 0.199 (-0.092, 0.489) | 0.179 | - | - |

^a^ Model 1 is univariate.

^b^ Model 2 is univariate (adjusted for age, sex, education, APOE ε4 carrier status, and clinical diagnosis).

^c^ Model 3 is step-wise multivariable regression model (adjusted for age, sex, education, APOE ε4 carrier status, and clinical diagnosis).

**Supplementary Table 11. Association between anxiety with AD biomarkers and CSVD markers in whole sample. [E]**

|  | Model 1^a^ | | Model 2^b^ | | Model 3^c^ | |
| --- | --- | --- | --- | --- | --- | --- |
|  | Estimate (95% CI) | *P* | Estimate (95% CI) | *P* | Estimate (95% CI) | *P* |
| Age | -0.012 (-0.028, 0.004) | 0.156 | - | - | - | - |
| Sex | 0.152 (-0.099, 0.403) | 0.236 | - | - | - | - |
| Education | -0.044 (-0.091, 0.004) | 0.070 | - | - | - | - |
| APOE ε4 carrier status | 0.160 (-0.091, 0.411) | 0.211 | - | - | - | - |
| Clinical diagnosis | 0.179 (-0.096, 0.454) | 0.202 | - | - | - | - |
| Aβ_42_ (pg/ml) | -0.003 (-0.006, 0.001) | **0.009** | -0.004 (-0.007, 0.001) | **0.015** | -0.004 (-0.007, -0.001) | **0.011** |
| p-tau_181_ (pg/ml) | 0.003 (-0.001, 0.008) | 0.142 | 0.002 (-0.002, 0.007) | 0.327 | - | - |
| t-tau (pg/ml) | 0.001 (-0.001, 0.003) | 0.339 | 0.001 (-0.002, 0.003) | 0.602 | - | - |
| p-tau_181_/Aβ_42_ | 0.502 (0.001, 1.003) | **0.049** | 0.410 (-0.145, 0.965) | 0.147 | - | - |
| t-tau/Aβ_42_ | 0.161 (-0.075, 0.397) | 0.181 | 0.136 (-0.133, 0.405) | 0.320 | - | - |
| PVL-WMH score | -0.122 (-0.271, 0.028) | 0.110 | -0.095 (-0.268, 0.079) | 0.286 | - | - |
| DWM-WMH score | -0.027 (-0.172, 0.119) | 0.719 | 0.014 (-0.150, 0.177) | 0.871 | - | - |
| BG-PVS score | 0.124 (-0.151, 0.398) | 0.376 | 0.151 (-0.124, 0.426) | 0.282 | - | - |
| CSO-PVS score | -0.104 (-0.268, 0.059) | 0.210 | -0.068 (-0.234, 0.98) | 0.421 | - | - |
| Microbleeds | 0.142 (-0.169, 0.453) | 0.370 | 0.185 (-0.147, 0.516) | 0.274 | - | - |
| Lacune | -0.047 (-0.365, 0.271) | 0.770 | -0.019 (-0.345, 0.308) | 0.910 | - | - |

^a^ Model 1 is univariate.

^b^ Model 2 is univariate (adjusted for age, sex, education, APOE ε4 carrier status, and clinical diagnosis).

^c^ Model 3 is step-wise multivariable regression model (adjusted for age, sex, education, APOE ε4 carrier status, and clinical diagnosis).

**Supplementary Table 12. Association between elation with AD biomarkers and CSVD markers in whole sample. [F]**

|  | Model 1^a^ | | Model 2^b^ | | Model 3^c^ | |
| --- | --- | --- | --- | --- | --- | --- |
|  | Estimate (95% CI) | *P* | Estimate (95% CI) | *P* | Estimate (95% CI) | *P* |
| Age | -0.001 (-0.008, 0.006) | 0.808 | - | - | - | - |
| Sex | 0.047 (-0.058, 0.152) | 0.378 | - | - | - | - |
| Education | -0.006 (-0.026, 0.013)0 | 0.532 | - | - | - | - |
| APOE ε4 carrier status | 0.004 (-0.101, 0.108) | 0.943 | - | - | - | - |
| Clinical diagnosis | 0.003 (-0.111, 0.118) | 0.955 | - | - | - | - |
| Aβ_42_ (pg/ml) | 0.000 (-0.001, 0.001) | 0.619 | 0.000 (-0.002, 0.001) | 0.580 | - | - |
| p-tau_181_ (pg/ml) | -0.001 (-0.003, 0.001) | 0.342 | -0.001 (-0.003, 0.001) | 0.327 | - | - |
| t-tau (pg/ml) | 0.000 (-0.001, 0.000) | 0.362 | 0.000 (-0.002, 0.001) | 0.365 | - | - |
| p-tau_181_/Aβ_42_ | -0.063 (-0.272, 0.146) | 0.556 | -0.075 (-0.309, 0.159) | 0.529 | - | - |
| t-tau/Aβ_42_ | -0.022 (-0.120, 0.077) | 0.661 | -0.021 (-0.135, 0.092) | 0.711 | - | - |
| PVL-WMH score | 0.021 (-0.042, 0.083) | 0.516 | 0.036 (-0.037, 0.109) | 0.332 | - | - |
| DWM-WMH score | -0.005 (-0.065, 0.056) | 0.883 | 0.000 (-0.069, 0.069) | 0.997 | - | - |
| BG-PVS score | -0.089 (-0.203, 0.025) | 0.127 | -0.089 (-0.205, 0.027) | 0.131 | - | - |
| CSO-PVS score | -0.066 (-0.134, 0.002) | 0.056 | -0.065 (-0.135, 0.004) | 0.066 | - | - |
| Microbleeds | 0.078 (-0.051, 0.208) | 0.235 | 0.097 (-0.042, 0.237) | 0.171 | - | - |
| Lacune | 0.062 (-0.070, 0.194) | 0.356 | 0.071 (-0.067, 0.208) | 0.313 | - | - |

^a^ Model 1 is univariate.

^b^ Model 2 is univariate (adjusted for age, sex, education, APOE ε4 carrier status, and clinical diagnosis).

^c^ Model 3 is step-wise multivariable regression model (adjusted for age, sex, education, APOE ε4 carrier status, and clinical diagnosis).

**Supplementary Table 13. Association between apathy with AD biomarkers and CSVD markers in whole sample. [G]**

|  | Model 1^a^ | | Model 2^b^ | | Model 3^c^ | |
| --- | --- | --- | --- | --- | --- | --- |
|  | Estimate (95% CI) | *P* | Estimate (95% CI) | *P* | Estimate (95% CI) | *P* |
| Age | 0.023 (0.001, 0.045) | **0.039** | - | - | - | - |
| Sex | 0.469 (-0.129, 0.810) | **0.007** | - | - | - | - |
| Education | -0.075 (-0.139, -0.010) | **0.023** | - | - | - | - |
| APOE ε4 carrier status | -0.050 (-0.393, 0.293) | 0.774 | - | - | - | - |
| Clinical diagnosis | 0.842 (0.475, 1.210) | **<0.001** | - | - | - | - |
| Aβ_42_ (pg/ml) | -0.004 (-0.007, 0.000) | **0.042** | -0.002 (-0.006, 0.002) | 0.325 | - | - |
| p-tau_181_ (pg/ml) | 0.001 (-0.005, 0.007) | 0.753 | -0.001 (-0.007, 0.005) | 0.810 | - | - |
| t-tau (pg/ml) | 0.001 (-0.002, 0.004) | 0.403 | 0.000 (-0.003, 0.003) | 0.966 | - | - |
| p-tau_181_/Aβ_42_ | 0.321 (-0.365, 1.006) | 0.359 | 0.071 (-0.669, 0.811) | 0.851 | - | - |
| t-tau/Aβ_42_ | 0.170 (-0.153, 0.493) | 0.301 | 0.033 (-0.325, 0.391) | 0.856 | - | - |
| PVL-WMH score | 0.102 (-0.102, 0.306) | 0.325 | -0.009 (-0.241, 0.223) | 0.939 | - | - |
| DWM-WMH score | 0.130 (-0.068, 0.328) | 0.197 | 0.045 (-0.173, 0.263) | 0.686 | - | - |
| BG-PVS score | -0.031 (-0.405, 0.344) | 0.873 | -0.060 (-0.426, 0.307) | 0.750 | - | - |
| CSO-PVS score | -0.002 (-0.225, 0.221) | 0.986 | 0.031 (-0.190, 0.252) | 0.780 | - | - |
| Microbleeds | 0.498 (0.076, 0.921) | **0.021** | 0.490 (0.050, 0.929) | **0.029** | - | - |
| Lacune | 0.376 (-0.056, 0.808) | 0.088 | 0.199 (-0.235, 0.634) | 0.368 | - | - |

^a^ Model 1 is univariate.

^b^ Model 2 is univariate (adjusted for age, sex, education, APOE ε4 carrier status, and clinical diagnosis).

^c^ Model 3 is step-wise multivariable regression model (adjusted for age, sex, education, APOE ε4 carrier status, and clinical diagnosis).

**Supplementary Table 14. Association between disinhibition with AD biomarkers and CSVD markers in whole sample. [H]**

|  | Model 1^a^ | | Model 2^b^ | | Model 3^c^ | |
| --- | --- | --- | --- | --- | --- | --- |
|  | Estimate (95% CI) | *P* | Estimate (95% CI) | *P* | Estimate (95% CI) | *P* |
| Age | -0.005 (-0.016, 0.007) | 0.433 | - | - | - | - |
| Sex | 0.221 (-0.046, 0.396) | **0.014** | - | - | - | - |
| Education | -0.044 (-0.077, 0.011) | **0.009** | - | - | - | - |
| APOE ε4 carrier status | 0.011 (-0.165, 0.187) | 0.904 | - | - | - | - |
| Clinical diagnosis | 0.187 (-0.005, 0.379) | 0.057 | - | - | - | - |
| Aβ_42_ (pg/ml) | 0.000 (-0.001, 0.002) | 0.617 | 0.001 (-0.001, 0.003) | 0.282 | - | - |
| p-tau_181_ (pg/ml) | 0.002 (-0.001, 0.005) | 0.213 | 0.002 (-0.002, 0.005) | 0.298 | - | - |
| t-tau (pg/ml) | 0.001 (-0.001. 0.002) | 0.423 | 0.001 (-0.001, 0.002) | 0.449 | - | - |
| p-tau_181_/Aβ_42_ | 0.137 (-0.215, 0.489) | 0.445 | 0.090 (-0.294, 0.475) | 0.645 | - | - |
| t-tau/Aβ_42_ | -0.071 (-0.237, 0.095) | 0.400 | -0.109 (-0.295, 0.077) | 0.250 | - | - |
| PVL-WMH score | -0.007 (-0.112, 0.098) | 0.893 | 0..023 (-0.098, 0.143) | 0.710 | - | - |
| DWM-WMH score | -0.043 (-0.144, 0.059) | 0.407 | -0.034 (-0.147, 0.080) | 0.559 | - | - |
| BG-PVS score | -0.181 (-0.372, 0.011) | 0.065 | -0.177 (-0.367, 0.013) | 0.067 | - | - |
| CSO-PVS score | -0.094 (-0.208, 0.020) | 0.107 | -0.067 (-0.182, 0.048) | 0.252 | -0.123 (-0.227, -0.019) | **0.021** |
| Microbleeds | 0.241 (0.024, 0.457) | **0.030** | 0.294 (0.066, 0.522) | **0.012** | 0.229 (0.022, 0.436) | **0.030** |
| Lacune | 0.058 (-0.165, 0.281) | 0.609 | 0.061 (-0.165, 0.287) | 0.596 | - | - |

^a^ Model 1 is univariate.

^b^ Model 2 is univariate (adjusted for age, sex, education, APOE ε4 carrier status, and clinical diagnosis).

^c^ Model 3 is step-wise multivariable regression model (adjusted for age, sex, education, APOE ε4 carrier status, and clinical diagnosis).

**Supplementary Table 15. Association between irritability with AD biomarkers and CSVD markers in whole sample. [I]**

|  | Model 1^a^ | | Model 2^b^ | | Model 3^c^ | |
| --- | --- | --- | --- | --- | --- | --- |
|  | Estimate (95% CI) | *P* | Estimate (95% CI) | *P* | Estimate (95% CI) | *P* |
| Age | 0.014 (-0.007, 0.034) | 0.189 | - | - | - | - |
| Sex | 0.661 (0.350, 0.972) | **<0.001** | - | - | - | - |
| Education | -0.040 (-0.100 ,0.019) | 0.185 | - | - | - | - |
| APOE ε4 carrier status | -0.188 (-0.504, 0.128) | 0.244 | - | - | - | - |
| Clinical diagnosis | 0.303 (-0.043, 0.649) | 0.086 | - | - | - | - |
| Aβ_42_ (pg/ml) | -0.001 (-0.004, 0.003) | 0.734 | 0.000 (-0.004, 0.003) | 0.884 | - | - |
| p-tau_181_ (pg/ml) | -0.001 (-0.006, 0.005) | 0.812 | 0.000 (-0.005, 0.06) | 0.889 | - | - |
| t-tau (pg/ml) | 0.000 (-0.003, 0.002) | 0.835 | 0.001 (-0.003, 0.004) | 0.740 | - | - |
| p-tau_181_/Aβ_42_ | 0.109 (-0.524, 0.743) | 0.735 | 0.256 (-0.433, 0.946) | 0.465 | - | - |
| t-tau/Aβ_42_ | 0.047 (-0.251, 0.346) | 0.756 | 0.150 (-0.183, 0.484) | 0.376 | - | - |
| PVL-WMH score | 0.124 (-0.064, 0.312) | 0.197 | 0.123 (-0.092, 0.339) | 0.262 | - | - |
| DWM-WMH score | 0.042 (-0.140, 0.225) | 0.649 | 0.026 (-0.177, 0.229) | 0.799 | - | - |
| BG-PVS score | -0.047 (-0.393, 0.299) | 0.790 | -0.057 (-0.399, 0.285) | 0.744 | - | - |
| CSO-PVS score | -0.036 (-0.242, 0.170) | 0.733 | -0.013 (-0.219, 0.193) | 0.901 | - | - |
| Microbleeds | 0.497 (0.107, 0.886) | **0.013** | 0.591 (0.183, 0.999) | **0.005** | 0.650 (0.232, 1.069) | **0.002** |
| Lacune | 0.392 (-0.007, 0.790) | 0.054 | 0.336 (-0.068, 0.740) | 0.103 | - | - |

^a^ Model 1 is univariate.

^b^ Model 2 is univariate (adjusted for age, sex, education, APOE ε4 carrier status, and clinical diagnosis).

^c^ Model 3 is step-wise multivariable regression model (adjusted for age, sex, education, APOE ε4 carrier status, and clinical diagnosis).

**Supplementary Table 16. Association between aberrant motor behavior with AD biomarkers and CSVD markers in whole sample. [J]**

|  | Model 1^a^ | | Model 2^b^ | | Model 3^c^ | |
| --- | --- | --- | --- | --- | --- | --- |
|  | Estimate (95% CI) | *P* | Estimate (95% CI) | *P* | Estimate (95% CI) | *P* |
| Age | 0.006 (-0.006, 0.017) | 0.327 | - | - | - | - |
| Sex | -0.050 (-0.224, 0.124) | 0.572 | - | - | - | - |
| Education | -0.031 (-0.064, 0.002) | 0.061 | - | - | - | - |
| APOE ε4 carrier status | -0.024 (-0.198, 0.150) | 0.788 | - | - | - | - |
| Clinical diagnosis | 0.403 (-0.216, 0.590) | **<0.001** | - | - | - | - |
| Aβ_42_ (pg/ml) | -0.002 (-0.003, 0.000) | 0.081 | -0.001 (-0.003, 0.001) | 0.294 | - | - |
| p-tau_181_ (pg/ml) | 0.004 (0.001, 0.007) | **0.008** | 0.003 (0.000, 0.006) | 0.054 | 0.003 (0.000, 0.007) | **0.042** |
| t-tau (pg/ml) | 0.002 (0.000, 0.003) | **0.021** | 0.001 (-0.001, 0.003) | **0.247** | - | - |
| p-tau_181_/Aβ_42_ | 0.472 (0.126, 0.817) | **0.008** | 0.368 (-0.011, 0.748) | **0.057** | - | - |
| t-tau/Aβ_42_ | 0.184 (0.021, 0.347) | **0.027** | 0.104 (-0.081, 0.288) | **0.269** | - | - |
| PVL-WMH score | 0.075 (-0.028, 0.178) | 0.155 | 0.044 (-0.076, 0.163) | 0.473 | - | - |
| DWM-WMH score | 0.080 (-0.020, 0.180) | 0.118 | 0.051 (-0.061, 0.164) | 0.368 | - | - |
| BG-PVS score | 0.045 (-0.145, 0.235) | 0.641 | 0.030 (-0.159, 0.219) | 0.754 | - | - |
| CSO-PVS score | 0.017 (-0.096, 0.131) | 0.764 | 0.032 (-0.082, 0.146) | 0.579 | - | - |
| Microbleeds | 0.078 (-0.138, 0.293) | 0.479 | 0.044 (-0.184, 0.271) | 0.706 | - | - |
| Lacune | 0.225 (0.006, 0.444) | **0.044** | 0.160 (-0.063, 0.384) | 0.159 | - | - |

^a^ Model 1 is univariate.

^b^ Model 2 is univariate (adjusted for age, sex, education, APOE ε4 carrier status, and clinical diagnosis).

^c^ Model 3 is step-wise multivariable regression model (adjusted for age, sex, education, APOE ε4 carrier status, and clinical diagnosis).

**Supplementary Table 17. Association between night-time behavior with AD biomarkers and CSVD markers in whole sample. [K]**

|  | Model 1^a^ | | Model 2^b^ | | Model 3^c^ | |
| --- | --- | --- | --- | --- | --- | --- |
|  | Estimate (95% CI) | *P* | Estimate (95% CI) | *P* | Estimate (95% CI) | *P* |
| Age | 0.001 (-0.024, 0.025) | 0.961 | - | - | - | - |
| Sex | 0.003 (-0.378, 0.385) | 0.986 | - | - | - | - |
| Education | -0.032 (-0.104, 0.039) | 0.377 | - | - | - | - |
| APOE ε4 carrier status | -0.048 (-0.429, 0.332) | 0.803 | - | - | - | - |
| Clinical diagnosis | -0.263 (-0.679, 0.153) | 0.215 | - | - | - | - |
| Aβ_42_ (pg/ml) | 0.001 (-0.003, 0.005) | 0.638 | 0.000 (-0.004, 0.005) | 0.899 | - | - |
| p-tau_181_ (pg/ml) | -0.003 (-0.010, 0.004) | 0.378 | -0.002 (-0.009, 0.005) | 0.528 | - | - |
| t-tau (pg/ml) | -0.001 (-0.004, 0.002) | 0.614 | 0.000 (-0.004, 0.003) | 0.911 | - | - |
| p-tau_181_/Aβ_42_ | -0.370 (-1.131, 0.390) | 0.339 | -0.284 (-1.134, 0.566) | 0.512 | - | - |
| t-tau/Aβ_42_ | -0.123 (-0.481, 0.236) | 0.501 | -0.066 (-0.478, 0.345) | 0.752 | - | - |
| PVL-WMH score | -0.032 (-0.258, 0.195) | 0.784 | -0.048 (-0.314, 0.218) | 0.724 | - | - |
| DWM-WMH score | -0.014 (-0.205, 0.234) | 0.897 | 0.010 (-0.241, 0.261) | 0.937 | - | - |
| BG-PVS score | -0.038 (-0.454, 0.378) | 0.859 | -0.062 (-0.483, 0.360) | 0.774 | - | - |
| CSO-PVS score | -0.216 (-0.463, 0.031) | 0.087 | -0.246 (-0.499, 0.007) | 0.057 | - | - |
| Microbleeds | 0.338 (-0.133, 0.808) | 0.159 | 0.459 (-0.047, 0.965) | 0.075 | 0.552 (0.050, 1.055) | **0.031** |
| Lacune | 0.016 (-0.465, 0.497) | 0.948 | 0.037 (-0.463, 0.536) | 0.885 | - | - |

^a^ Model 1 is univariate.

^b^ Model 2 is univariate (adjusted for age, sex, education, APOE ε4 carrier status, and clinical diagnosis).

^c^ Model 3 is step-wise multivariable regression model (adjusted for age, sex, education, APOE ε4 carrier status, and clinical diagnosis).

**Supplementary Table 18. Association between appetite with AD biomarkers and CSVD markers in whole sample. [L]**

|  | Model 1^a^ | | Model 2^b^ | | Model 3^c^ | |
| --- | --- | --- | --- | --- | --- | --- |
|  | Estimate (95% CI) | *P* | Estimate (95% CI) | *P* | Estimate (95% CI) | *P* |
| Age | 0.004 (-0.018, 0.025) | 0.732 | - | - | - | - |
| Sex | 0.356 (0.029, 0.684) | **0.033** | - | - | - | - |
| Education | -0.041 (-0.102, 0.021) | 0.198 | - | - | - | - |
| APOE ε4 carrier status | -0.125 (-0.453, 0.203) | 0.454 | - | - | - | - |
| Clinical diagnosis | 0.806 (0.454, 1.158) | **<0.001** | - | - | - | - |
| Aβ_42_ (pg/ml) | -0.001 (-0.005, 0.002) | 0.449 | -0.001 (-0.004, 0.004) | 0.960 | - | - |
| p-tau_181_ (pg/ml) | 0.002 (-0.004, 0.008) | 0.472 | 0.001 (-0.005, 0.007) | 0.844 | - | - |
| t-tau (pg/ml) | 0.002 (0.000, 0.005) | 0.103 | 0.001 (-0.002, 0.004) | 0.358 | - | - |
| p-tau_181_/Aβ_42_ | 0.195 (-0.462, 0.851) | 0.560 | -0.034 (-0.747, 0.679) | 0.925 | - | - |
| t-tau/Aβ_42_ | 0.123 (-0.187, 0.432) | 0.436 | 0.024 (-0.321, 0.369) | 0.891 | - | - |
| PVL-WMH score | -0.028 (-0.223, 0.167) | 0.778 | -0.062 (-0.285, 0.161) | 0.583 | - | - |
| DWM-WMH score | -0.017 (-0.207, 0.172) | 0.857 | -0.037 (-0.247, 0.173) | 0.732 | - | - |
| BG-PVS score | -0.002 (-0.361, 0.357) | 0.992 | 0.008 (-0.346, 0.361) | 0.965 | - | - |
| CSO-PVS score | -0.134 (-0.347, 0.080) | 0.219 | -0.075 (-0.288, 0.138) | 0.489 | - | - |
| Microbleeds | 0.342 (-0.064, 0.748) | 0.098 | 0.350 (-0.074, 0.774) | 0.106 | - | - |
| Lacune | 0.244 (-0.171, 0.659) | 0.248 | -/156 (-0.263, 0.575) | 0.464 | - | - |

^a^ Model 1 is univariate.

^b^ Model 2 is univariate (adjusted for age, sex, education, APOE ε4 carrier status, and clinical diagnosis).

^c^ Model 3 is step-wise multivariable regression model (adjusted for age, sex, education, APOE ε4 carrier status, and clinical diagnosis).
